# A Web-Based Alcohol Screening and Brief Intervention Training Module Within Physician Assistant Programs in the Midwest to Increase Knowledge, Attitudes, and Confidence: Evaluation Study

**DOI:** 10.2196/11963

**Published:** 2019-10-23

**Authors:** Leigh E Tenkku Lepper, Tracy Cleveland, Genevieve DelRosario, Katherine Ervie, Catherine Link, Lara Oakley, Abdelmoneim Elfagir, Debra J Sprague

**Affiliations:** 1 School of Social Work and Public Health Program University of Missouri-Columbia Columbia, MO United States; 2 Physician Assistant Program University of South Dakota Vermillion, SD United States; 3 Physician Assistant Program Saint Louis University St Louis, MO United States; 4 Physician Assistant Program University of Missouri-Kansas City Kansas City, MO United States; 5 Missouri Institute of Mental Health University of Missouri-St Louis St Louis, MO United States

**Keywords:** alcohol education, alcohol screening and brief intervention, Web-based training, standardized patient, physician assistant

## Abstract

**Background:**

Preventing and reducing risky alcohol use and its side effects remains a public health priority. Discussing alcohol use with patients can be difficult; dedicated training for health care providers is needed to facilitate these conversations. A Web-based alcohol screening and brief intervention (SBI), comprising didactic and skills application training, was designed for physician assistant students.

**Objective:**

This paper details experiences and outcomes in developing an alcohol SBI training curriculum and coordinating virtual encounters with standardized patients. We also explain challenges faced with developing an alcohol SBI training and a Web-based learning management site to fit the needs of 5 different physician assistant programs.

**Methods:**

Training development comprised 3 phases—precourse, development, and implementation. The precourse phase included developing the initial training curriculum, building a website, and testing with a pilot group. The development phase refined the training curriculum based on user feedback and moved into a three-component module: didactic training module, guided interactive encounter with a simulated patient, and live encounter with a standardized patient. A learning management system website was also created. In the implementation phase, 5 physician assistant schools incorporated the Web-based training into curricula. Each school modified the implementation method to suit their organizational environment. Evaluation methods included pre- and postchange over time on trainee attitudes, knowledge, and skills (confidence) on talking to patients about alcohol use, trainee self-reported proficiency on the standardized patient encounter, standardized patient evaluation of the trainee proficiency during the alcohol use conversation, user evaluation of the type of technology mode for the standardized patient conversation, and overall trainee satisfaction with the Web-based training on alcohol SBI.

**Results:**

Final evaluation outcomes indicated a significant (*P*<.01) change over time in trainee knowledge and skills (confidence) in the conduct of the alcohol SBI with a standardized patient, regardless of the program implementation method. Trainees were generally satisfied with the Web-based training experience and rated the use of the videoconference medium as most useful when conducting the alcohol SBI conversation with the standardized patient. Training that included a primer on the importance of screening, individual participation in the Web-based didactic alcohol SBI modules, and virtual encounters with standardized patients through a university-based simulation center was the most widely accepted. Successful implementation included program investment and curriculum planning. Implementation barriers involved technical challenges with standardized patient encounters and simulation center logistics, and varying physician assistant school characteristics.

**Conclusions:**

Development and implementation of Web-based educational modules to educate health care professionals on alcohol SBI is effective, easy to reproduce, and readily accessible. Identifying challenges affecting development, implementation, and utilization of learned techniques in practice, enhances facilitation of learning and training efficacy. As the value of technology-based learning becomes more apparent, reports detailing what has worked versus what has not may help guide the process.

## Introduction

I will have patients that will need alcohol counseling, and this [alcohol SBI training] helped me to approach that subject in a better way with future patients.

Physician Assistant student 2016 pilot

Numerous studies have found that health care providers, especially physicians, lack the knowledge and confidence to inquire about patient alcohol use behavior. Primary care professionals remain uncomfortable when talking to their patients about alcohol use [1-4]. Screening, brief intervention, and referral to treatment (SBIRT) is an evidence-based, public health approach utilized to screen patients and initiate a conversation on alcohol use, which may lead to a brief intervention. This method is recommended by the Institute of Medicine, the American College of Obstetricians and Gynecologists, the Centers for Disease Control and Prevention, the Substance Abuse and Mental Health Services Administration (SAMHSA), and many other research, policy, and public health organizations to reduce alcohol exposure and alcohol use disorders with demonstrated effectiveness [5]. However, challenges remain with the diffusion of SBIRT principles into routine practice. Research suggests finding more creative and engaging ways to teach the principles of SBIRT to ensure that primary care providers (PCPs) routinely utilize this technique in practice [6]. Therefore, a key translational research question to answer is how to address training gaps in advancing SBIRT knowledge and utilization.

As part of coordinated efforts to advance the utilization and adoption of SBIRT in primary care, researchers have focused on developing new and improved ways of teaching SBIRT to PCPs. Some of these methods have included utilizing Web-based training models [7], incorporating training materials into health care provider curricula [8,9], engaging simulated patients [10], and creating Web-based interactive platforms [11]. Although mixed results have been reported on the effectiveness of these approaches in health care education, interventions that include interactivity, continued practice exercises, repetition, and feedback appear to improve learning outcomes [12]. A recent report found that the referral to treatment component of SBIRT had not been fully implemented within practice settings and, therefore, had not been shown as effective, compared with the screening and brief intervention (SBI) components [13]. The focus of this training for the physician assistant has been on primary care screening for all patients, with greater emphasis placed on alcohol SBI as an equally effective first line of prevention for identifying at-risk alcohol use.

With the evolution of the internet as a tool for instruction, Web-based methods have been acclaimed in the literature for their benefits in academic training. These benefits, including convenience, reach, and availability, have continued to expand, providing limitless opportunities for academic development [12]. As benefits of Web-based learning for health care students have become more apparent, researchers have begun to explore the feasibility of incorporating these approaches to advance training and utilization of SBIRT in practice settings [14]. Mixed results have also been reported on the effectiveness of these approaches in terms of presenting the training material, engaging participants, and ensuring that trainees utilize this knowledge in clinical practice settings [6,15]. Further research is required to understand what aspects of Web-based trainings resonate with participants and are more likely to be helpful in continued practice.

Engaging standardized patients in the training of health care professionals is another training approach that is widely supported by the literature. Standardized patients are individuals from the community who have been trained to consistently portray patient roles and role-play health states typically found in health care practices. Evidence from several studies suggests that engaging the standardized patient in health care professional training can be beneficial in many ways, including building interactivity, collecting patient histories, building confidence, providing realistic practice scenarios within a safe space, and assessing student level of skill acquisition [9,16,17]. Some studies also suggest that SBIRT skills are reinforced when standardized patients work with health care professionals [8-10]. In their study, Lempicki et al [11] found that interprofessional teams participating in a videoconference encounter with a standardized patient enjoyed the encounter more than those participating in face-to-face encounters. Although this finding did not translate into continued utilization of this training resource, it might suggest avenues for more research exploration [11].

With the increased uptake of electronic learning (e-learning) approaches in health professional academic training, there is a need to understand what factors influence the development, implementation, and sustainability of e-learning approaches with the goal of increasing diffusion of alcohol SBI techniques. Similarly, as the benefits of utilizing standardized patients in health care professional training continually emerge, there is a need to explore, identify, and strengthen the aspects of standardized patient encounters found most beneficial to trainees, particularly for alcohol SBI. In the Midwestern United States, many physician assistant schools have not implemented any alcohol screening training in their curriculum. Consequently, upon graduation, students may not have acquired the necessary skills to start, maintain, and conclude conversations around alcohol use with their patients. With the growing capacity—in breadth of content matter and rigor and increasing number of students—of these programs and the time required to train and learn the elements of alcohol SBI, there is an increased need to deliver training using Web-based approaches. This study sought to incorporate both e-learning approaches and standardized patients to deliver and practice SBI techniques for alcohol use. We hypothesized that combining Web-based alcohol SBI approaches and video conferencing with standardized patients would improve learning and utilization of these techniques in practice settings. In this paper, we report on lessons learned while designing a Web-based didactic alcohol SBI course that included a guided interactive experience with 2-character scenarios and virtual live encounters with a standardized patient for physician assistants, incorporated into already established student curricula. We also report on how the training was adapted to address the unique contextual factors of each academic program and challenges faced with implementing the program. And finally, we report the evaluation outcomes of the change in attitudes, knowledge, and skills in terms of confidence of trainees along with satisfaction of using e-learning methods for teaching physician assistants how to talk with their patients about alcohol use.

## Methods

### Phase 1: Precourse Development

In the original proposal, we had planned to develop the alcohol SBI education training curriculum for the physician assistant in 2 parts: a didactic component, based upon SBIRT training slides from the SAMHSA Ideas Exchange, and an experiential component, designed to be used with an avatar for virtual communication using Second Life (a platform used for avatar-based video gaming), accessed via downloadable apps. The initial version of the didactic training module was completed within the first 4 months of the 3-year grant period and submitted for faculty feedback in February 2016. The pilot cohort of physician assistant students completed the training in March 2016. In addition to the alcohol SBI education module, we created a separate SBI implementation into practice module, a learning management system (LMS) website named Catalyst Learning Center [18], and the Catalyst Central virtual world avatar experience in the first 6 months of the study.

Feedback from the first cohort of student trainees provided information on the utility, success, and challenges of learning and practicing alcohol SBI in a Web-based format. Students were asked to respond to the question, what about the training was most useful in supporting your work responsibilities? Positive qualitative comments from student experiences included:

It provided useful tips for communicating difficult topic areas. Also, there were several small details and beneficial pneumonics that helped to remember some of the subject [material].

University of Missouri–Kansas City (UMKC) 68/2016

I will have patients that will need alcohol counseling and this helped me to approach that subject in a better way with future patients.

UMKC80/2016

A simulation satisfaction survey also solicited feedback regarding the aspects of the training for improvement, inclusive of curriculum, and modality. Responses included:

I think that you should throw out the avatar idea altogether. I think it's more useful to look directly at a real person than do the avatar anyway. There were so many glitches with that avatar. That was somewhat frustrating.

UMKC65/2016

The training modules were very wordy…Also, I think more emphasis needs to be placed on how to proceed through the actual interview itself…when to use the ten questions from the Alcohol Use Disorders Identification Test interview and how to introduce the topic to the patient gently without offending them.

UMKC70/2016

The use of the Second Life platform proved to be significantly challenging for students to access and, based upon their feedback, we discontinued the use of Second Life. Subsequently, we chose to build our own experiential platform that we believed would ultimately increase the likelihood of a successful and effective experience.

### Phase 2: Development

On the basis of initial feedback, the team made several significant changes to the alcohol SBI training curriculum. Revisions included a completely redesigned Web-based training course, alcohol SBI training for the physician assistant, with 3 components: a didactic module, an experiential module with a guided interactive alcohol SBI encounter attached to the didactic training, and a live encounter using videoconferencing with a standardized patient. The entire training course was housed at Catalyst Learning Center [18], and all users had to register to participate. The didactic module is narrated and interactive, covering the elements of screening and brief intervention; motivational interviewing; alcohol use among adults, teens, and pregnant women; and appropriate screening tools and how to use them.

**Figure 1:**
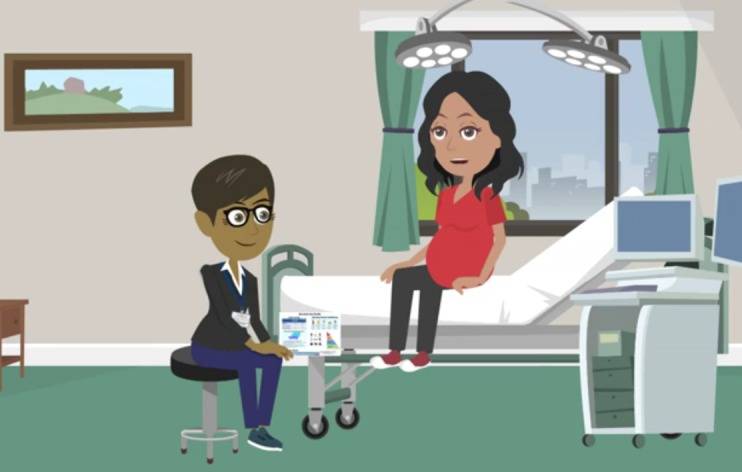
Avatar-like character on the Catalyst Learning Center website walking trainees through alcohol screening and brief intervention training.

**Figure 2:**
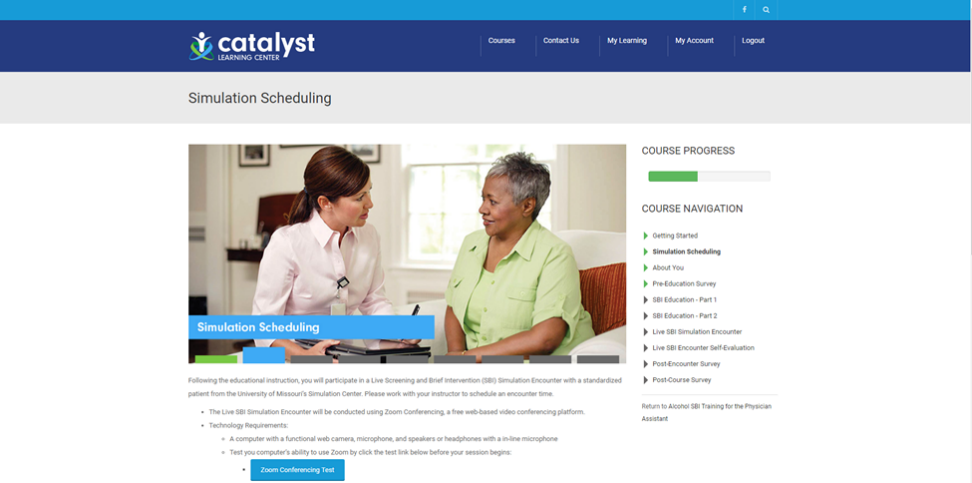
Simulation scheduling through Catalyst Learning Center website.

The guided interactive alcohol SBI encounter included avatar-like characters and allowed the student to immediately practice the alcohol SBI skills learned during the preceding didactic course. A patient scenario is presented along with scripted options in drop-down menus (Figure 1). The trainee proceeds through a scripted alcohol SBI patient encounter and must apply technical skills, such as implementing the appropriate screening tool, understanding the format of a brief intervention, and using interpersonal skills. The third segment of the training included a scheduled live encounter with a standardized patient at a university-based simulation center using videoconferencing software (Figure 2).

#### Trainee Process

The full Web-based training course comprised a live-encounter scheduling process, pretraining evaluations, the didactic module, 2 guided interactive alcohol SBI encounters, the live alcohol SBI encounter with a standardized patient via video conference, and posttraining evaluations. At 30 days posttraining, an email was sent to the students requesting an additional follow-up evaluation to be completed at the Catalyst website [18].

#### Development and Implementation of the Catalyst Learning Center Learning Management System

An LMS website, Catalyst Learning Center [18], was designed by contractors hired for this study. The LMS captures data on the training course, securely houses personal information, and provides certificates of completion for continuing medical education and continuing educatioN units for a wide variety of professionals. We had a contract with the Creighton University Continuing Education Department to provide approved certification of the continuing medical education and continuing education credits for professionals.

### Phase 3: Implementation

The alcohol SBI training program was implemented in 3 accredited physician assistant programs and in a new physician assistant program in the state of Missouri and a well-established physician assistant program at South College in Knoxville (SC-Knoxville), Tennessee. The entire training program was implemented in 3 stages: (1) a precourse stage in which faculty were trained, who then taught students about the importance of alcohol screening; (2) a Web-based alcohol use didactic course plus a guided interactive alcohol SBI encounter with a simulated avatar-like patient; and (3) a face-to-face encounter with a standardized patient using videoconferencing. In one physician assistant program, a translation into practice stage was introduced with preceptors in which preceptors were taught how to integrate the training into the student curriculum. To begin training, students received guidance from the research team and their academic instructor to participate in the Web-based training module. This was followed by a discrete link provided by the academic instructor connecting students to a cohort-specific landing page at Catalyst Learning Center [18], prompting students to register and automatically enroll in the designated alcohol SBI course section. Students were given the opportunity to self-schedule for the live virtual encounter with the standardized patient ahead of a prescribed deadline and prearranged live-encounter date.

### Settings

The 5 physician assistant programs that agreed to participate in the development, implementation, and data collection for this sudy included the following: University of Missouri-Kansas City (UMKC), Missouri State University (MSU), Saint Louis University (SLU), Stephens College (SC-Columbia), and South College (SC-Knoxville). Table 1 provides the name and location of the academic program, a description of the academic program, and how the alcohol SBI training was implemented within each academic setting.

**Table 1:** Program and implementation descriptions of each physician assistant program.

Academic program	Program description	Implementation description
University of Missouri-Kansas City Master of Medical Science Physician Assistant Program (Kansas City, Missouri)	28-month program, created in 2014; urban setting; annually accepts 20 students	Alcohol SBI^a^ training program occurred in year 1, semester 1, and was included as part of *Physician Assistant Professions I* course; initial 2-hour lecture provided by instructors and included a workshop on essential traits of effective communication and discussion on importance of alcohol use screening; students were given 1 week outside of class to complete the alcohol SBI training on the Web and the live standardized patient encounter; final discussion held as debriefing to discuss student experience with the training and the live standardized patient encounter
Missouri State University Physician Assistant Studies Program (Springfield, Missouri)	24-month program, created in 2000; urban setting; annually accepts 30 students	Alcohol SBI training occurred in year 1, semester 1, and was included as part of the *Behavioral Medicine* course; faculty-held initial discussion on alcohol use; students were exposed to Web-based training over a 3-week period, utilizing 2 2-hour class sessions—week 1, alcohol SBI course completed on the Web; week 2, live encounter completed outside class; week 3, faculty debriefing session on student experience with Web-based training and live standardized patient encounter; during clinical year, alcohol SBI utilization was tracked using the E-value^b^ system
Saint Louis University Master of Medical Science, Physician Assistant Program (St Louis, Missouri)	27-month program, created in 1971; urban setting; annually accepts 46 students	Training occurred in 4th didactic semester before clinicals and was a part of the *Essential of Pediatrics* course; training participation was considered extra credit; faculty-held discussion provided preparation for the course; students completed alcohol SBI course independently and scheduled live standardized patient encounters; instructor debriefing session held during class on student perception of experience and utilization of training in subsequent clinical year
Stephens College Master of physician assistant Studies Program (Columbia, Missouri)	27-month program, began in August 2016; largely urban setting; annually accepts 20 students	Live presentation by the first author on role of Physician Assistant in OBGYN^c^ and FASD^d^ as part of the *OBGYN Clinical* module; students completed alcohol SBI course independently following live presentation; live standardized patient encounters conducted on campus in controlled environment to decrease technical challenges
South College Master of Health Science in Physician Assistant Studies Program (Knoxville)	27-month program, created in 2007; urban setting; annually accepts 85 students	Alcohol SBI training occurred in year 2 of the clinical year as part of the OBGYN Clinical module; no initial discussions held with students; live presentation by the first author on the role of a physician assistant in OBGYN and FASD; students completed alcohol SBI course independently before live standardized patient encounter, scheduled within 1 week of the in-class presentation

^a^SBI: screening and brief intervention.

^b^E-value: E-value is a Web-based evaluation system designed to help manage one’s medical education program.

^c^OBGYN: obstetrician-gynecologist.

^d^FASD: fetal alcohol spectrum disorder.

### Measurement Instruments

The evaluation plan was based on a set of questionnaires to assess the improvement in attitudes, knowledge, and skills (in terms of confidence) of trainees; how well the alcohol SBI encounter with the standardized patient occurred from the perspectives of the trainee and the standardized patient; a feedback survey on technological settings utilized for the training; and finally, a set of overall training satisfaction questions. All of the evaluation assessments were conducted on the Web using Qualtrics within the Catalyst Learning Center LMS [18] and included surveys and scales as follows.

#### Pre- and Posttest: Attitudes, Knowledge, and Skills Survey

Trainees completed a self-assessment on 9 attitude and knowledge statements and 5 skills (confidence) statements in the application of alcohol SBI techniques before and after the training. The attitudes, knowledge, and skills survey was scored using a Likert-type 7-point response scale, with 1=strongly disagree and 7=strongly agree with the given statement. The attitudes, knowledge, and skills survey was designed by the researchers at the UMKC. For this study, psychometric properties were measured with Cronbach alpha and revealed strong internal consistency (alpha=.80).

#### Baseline and 1-Month Follow-Up Satisfaction Surveys

A total of 4 items were adopted from the Center for Substance Abuse Treatment standard measurements of training satisfaction as required by the grant. The baseline and 1-month follow-up survey statements were scored using a Likert-type 5-point response scale, with 1=strongly disagree/dissatisfied and 5=strongly agree/satisfied with the given statement.

#### Proficiency Rating Scale–Provider

This scale was used to assess the live virtual alcohol SBI encounter between the provider (in this case, trainee) and the standardized patient, from the perspective of the trainee. A set of 11 statements assessed the trainee’s perception of how proficiently they applied the skills learned in the alcohol SBI training within the simulated patient encounter with a standardized patient. A 5-point response scale was used, with 1=I did not do this; 2=I attempted but could improve on skill/technique for best practice; 3=I performed this skill/technique at a level that is approaching acceptable; 4=I did this well, with good technique; and 5=I did very well, with positive reception and engagement from the patient. In addition, 2 qualitative questions requested trainee feedback on their perspective of how well they conducted the conversation with the standardized patient. The first question asked the trainee, “what 2 things did you like about the way you conducted this intervention?” and the second question asked “what 2 ways do you feel you could improve your skills in these conversations?”

#### Proficiency Rating Scale–Standardized Patient

This scale was used to assess the virtual alcohol SBI encounter between the provider/trainee and the standardized patient, from the perspective of the standardized patient. A set of 10 statements asked the standardized patient to rate the trainee’s proficiency in the simulated alcohol SBI encounter. A 5-point response scale was used, with 1=did not do this; 2=attempted, but could improve; 3=nearing acceptable skill; 4=done well; and 5=done very well. A separate question asked the standardized patient if “this conversation increased my motivation to cut down or quit drinking, or at least to consider doing so” and was assessed with a Likert-type scale ranging from 1=strongly disagree to 5=strongly agree. Finally, the standardized patient provided feedback on 2 positive observations about the way the trainee conducted the intervention and 2 ways the trainee could improve his/her skills in future conversations.

#### Telecom Simulation User Evaluation Survey

This survey asked the trainee to assess their experience with using the various types of technological settings in which to hold the alcohol SBI encounters. The three settings used included teleconference, avatar/virtual world, or a phone encounter when video conferencing was nonviable. The trainee responded to 12 statements that provided feedback on how well the use of technology fared compared with real-life or face-to-face encounters with a live standardized patient. The assessment utilized a Likert-type 5-point response scale ranging from 1=strongly disagree to 5=strongly agree. Finally, two qualitative questions asked the trainee to provide what they liked best about the training and what suggestions they had for improving the training.

### Assessment Time Intervals

Pre- and posttest attitudes, knowledge, and skills (in terms of confidence) assessments were completed immediately before and after the didactic Web-based training course. The baseline satisfaction survey was completed at the end of the entire course, whereas the 1-month follow-up satisfaction survey was completed, with a direct link sent to the student, 1 month post training. The Proficiency Rating Scale (PRS) was completed by both the trainee and the standardized patient immediately following the simulated patient encounter. The Telecom Simulation User Evaluation Survey was completed at the completion of the simulated patient encounter.

### Analytical Plan

All pre/posttest mean scores were examined using a *t* test for statistical significance. We also examined for differences between pre- and posttest assessments stratified by demographics. A mixed-models factorial analysis of variance (ANOVA) stratified by institution was used to assess any differences in terms of the training outcomes and effectiveness between institutions. Finally, we conducted a comparison of outcome characteristics between responders and nonresponders to evaluate the impact of attrition on our results. All analyses were conducted using SPSS version 25.0. The institutional review board of the UMKC reviewed and approved this evaluation study.

## Results

### Demographic Characteristics

Demographics of all physician assistant trainees who completed the training are shown in Table 2.

**Table 2:** Demographics of physician assistant trainees (N=482).

Demographic category		n (%)
**Gender**		
	Female	341 (70.7)
	Male	141 (29.3)
**Race**		
	White	437 (90.7)
	Black	15 (3.1)
	Asian	24 (5.0)
	Other	6 (1.2)
**Education**		
	4-year degree	431 (89.4)
	Master-level degree	48 (10.0)
	Doctoral-level degree	2 (0.4)
	Some college	1 (0.2)
**Year in program**		
	Didactic	264 (54.7)
	Clinical	218 (45.2)
**Academic institution**		
	Saint Louis University	54 (11.2)
	University of Missouri–Kansas City	72 (14.9)
	Missouri State University	91 (18.9)
	South College	227 (47.1)
	Stephens College	38 (7.9)

The majority of trainees were female 70.7% (341/482), white 90.7% (437/482), had completed a 4-year degree 89.4% (431/482), and were in the first year of their physician assistant program 54.7% (264/482).

### Knowledge, Attitude and Confidence Change Over Time

Table 3 provides the comparison between the pre- and postsurvey results, showing the change in attitudes, knowledge, and skills (in terms of confidence) of the trainees. With the exception of four of the nine attitude and knowledge statements, we saw significant differences (*P*<.01) in mean scores over time in attitudes, knowledge, and skills (in terms of confidence) level of the trainees after completing the Web-based alcohol SBI training course. The overall aggregate score across all 14 statements went from a mean score of 5.06 for the pretest survey to a mean score of 5.73 in the posttest survey (*P*<.01). For the statement “Learning to screen and intervene in patients with hazardous or harmful substance use is important for me in my current/future position”, the overall mean score for the pretest was 6.48 and decreased slightly to 6.39 (*P*=.70), indicating a nonsignificant difference. For the statement “Substance use and associated risk are not appropriate topics to address with patients in my current or future practice,” the mean score for the pretest was 6.15 and decreased slightly to 6.13 (*P*=.99), indicating a nonsignificant difference. Finally, the statement “There are many nonphysicians (social workers and others) I work with who address alcohol and drug problems skillfully” resulted in a mean score of 4.86 in the pretest survey and decreased slightly to 4.80 (*P*=.16) in the posttest survey, also indicating a nonsignificant relationship.

**Table 3:** Mean scores on knowledge, attitude, and confidence pre- and posttest survey (N=482).

Knowledge, attitude, and confidence questions	Pretest, mean (SD)	Posttest, mean (SD)	*P* value
I have a good understanding of alcohol and substance use	5.35 (1.01)	5.95 (1.04)	<.001
Learning to screen and intervene in patients with hazardous or harmful substance use is important for me in my current/future position	6.48 (0.77)	6.39 (1.09)	.70
Substance use and associated risks are not appropriate topics to address with patients in my current or future practice	6.15 (1.36)	6.13 (1.51)	.99
There are many physicians I work with who address alcohol and drug problems skillfully and effectively	4.72 (1.18)	4.59 (1.32)	.02
There are many nonphysician providers (social workers and others) I work with who address alcohol and drug problems skillfully	4.86 (1.16)	4.80 (1.24)	.16
I am confident in my ability to screen patients for alcohol/drug problems	4.22 (1.34)	5.70 (0.88)	<.001
I am confident in my ability to assess patients' readiness to change their behavior	4.59 (1.19)	5.81 (0.89)	<.001
I am confident in my ability to discuss patients’ substance use and advise them to change their behavior	4.48 (1.32)	5.78 (0.88)	<.001
I am confident in my ability to refer patients with alcohol/drug problems	4.78 (1.29)	5.77 (0.96)	<.001
It takes too much time to deal with the drinking/drug behavior of my patients	5.59 (1.26)	5.93 (1.18)	<.001
Patients will be angry if I ask questions about their substance use	4.03 (1.21)	5.06 (1.25)	<.001
My interaction with a patient can make a difference regarding their use of substances	5.84 (0.97)	6.23 (0.83)	<.001
Incorporating screening, brief intervention, and referral to treatment into routine medical practice is critical for meeting health care needs	5.52 (1.11)	6.14 (0.91)	<.001
I feel confident in my understanding of low-risk drinking limits	4.28 (1.33)	5.96 (0.85)	<.001
Aggregate scores	5.06 (0.61)	5.73 (0.56)	<.001

### Proficiency (Skill) Rating Scale Completed By the Trainee

The PRS-provider was completed by the trainee immediately following the alcohol SBI encounter with the standardized patient. Overall, the trainees scored themselves as conducting the conversation at a performance level *approaching acceptable* (mean 3.41, SD 0.69). We note that the students rated themselves low for statement number 3 in comparison with the other elements of the PRS-provider. This statement asks about the National Institute on Alcohol Abuse and Alcoholism (NIAAA) low-risk drinking guidelines that are discussed in the training module as one of several alcohol screening instruments. Quantitative results for the PRS-provider are presented in Table 4.

The final 2 questions on the PRS-provider (trainee) asked the trainee to respond to two open-ended questions in which they reflected on how well they believed they conducted their initial conversation with a patient about alcohol use. In response to the first question “What two things did you like about the way you conducted this intervention?” a student responded:

I feel that I was able to look beyond just the systemic health risks associated with their increased drinking and how cutting back can improve the occurrence of accidents that put them in danger.

SLU230/2016

Whereas a second student responded:

I felt as though I was able to have a conversation as opposed to just spitting facts at the patient.

SC-Columbia339/2017

In response to the second question “What two ways do you feel you could improve your skills in these conversations?” a student stated:

I need to improve my comfort level with discussing “harder” topics with patients. This was my first alcohol discussion so I felt more nervous and need to improve my confidence and comfort level.

MSU356/2017

Whereas another student responded:

I felt like I was talking a lot more than the patient. I was focused on making sure I got all my points across that I think I should have slowed down and allowed the patient to express her thoughts a little bit more.

SC-Knoxville540/2017

**Table 4:** Mean scores on the Proficiency Rating Scale–Provider (n=474).

Trainee self-reported skill level items	Mean (SD)
Ask for permission to talk about patient’s alcohol use	4.03 (0.78)
Assess quantity, frequency, and consequences of alcohol use	3.72 (0.85)
Explain the National Institute on Alcohol Abuse and Alcoholism low-risk drinking guidelines (including *0 drinks for pregnant women* and associated health risks)	2.56 (1.14)
Advise the patient to quit or cut down on alcohol use	3.74 (0.89)
Help the patient think about pros and cons of his/her alcohol use	3.19 (1.09)
Ask how ready s/he is to make a change	3.70 (0.96)
Help the patient make a plan or set a goal for decreasing use and/or discussing further	3.47 (1.01)
Explore patient’s own reasons for quitting or cutting down on alcohol use	3.01 (1.11)
Work with the patient as a partner in addressing his/her alcohol use issues	3.27 (1.02)
Support his/her autonomy and choice regarding alcohol use	3.49 (0.98)
Proficiency Rating Scale aggregate scores	3.41 (0.69)

### Proficiency (Skill) Rating Scale Completed By the Standardized Patient

In contrast, the PRS–standardized patient completed by the standardized patient scored the trainees at a somewhat higher performance (mean 3.77, SD 0.74). Results for the PRS–standardized patient are presented in Table 5.

For the PRS–standardized patient, the standardized patient responded to two open-ended questions, giving very detailed feedback to each trainee that might help improve their skills in having a conversation about alcohol use with their patients. In response to the first question “What two things did you like about the way the trainee conducted this intervention?”, a standardized patient responded:

When you used the statement, “bringing to your attention...” regarding my at-risk use, I felt respected by the nonconfrontational way of bringing this up.

Standardized patient for SC-Knoxville540/2017

Another standardized patient provided the following feedback:

I appreciated how this provider responded to the discrepancy of my former OB’s advice that a little alcohol in pregnancy was okay versus my current provider’s recommendation that no amount of alcohol is safe during pregnancy. The student validated the former physician’s recommendation by stating that “Maybe things have changed since your last pregnancy” and went on to communicate the current safe limits recommended now, which of course is no alcohol.

UMKC128/2016

For the second question “What two ways could this trainee improve his/her skills in these conversations?” a comment from a standardized patient to the trainee included:

When I asked if my glass of wine had hurt my baby, I LOVED the response, “Let’s just focus on moving forward.” It eliminated any guilt but also didn't give any false promises. It was a terrific way to handle that question.

UMKC72/2016

**Table 5:** Mean scores on the Proficiency Rating Scale–Standardized Patient (n=474).

Proficiency Rating Scale–Standardized Patient items	Mean (SD)
Asked for permission to talk about my alcohol use	4.00 (0.86)
Assessed quantity, frequency, and consequences of my alcohol use	4.01 (0.79)
Explained specific National Institute on Alcohol Abuse and Alcoholism low-risk drinking guidelines and health risks to me	3.66 (0.97)
Advised me to quit or cut down on alcohol use	3.78 (0.93)
Helped me think about pros and cons of my alcohol use	3.50 (1.16)
Asked how ready I am to make a change	3.77 (1.02)
Helped me make a plan or set a goal for decreasing (or quitting) my alcohol use	3.72 (1.09)
Explored my own possible reasons for quitting or cutting down on my alcohol use	3.40 (1.22)
Worked with me as a partner (respectfully and nonjudgmentally) in addressing my alcohol use issues	3.96 (0.94)
Supported my autonomy and choice regarding my alcohol use	3.97 (0.89)
Proficiency Rating Scale aggregate scores	3.77 (0.74)

### Telecom Simulation User Evaluation Survey

The Telecom Simulation User Evaluation Survey measured the user satisfaction with three different types of technology used for the alcohol SBI encounter with the standardized patient and are presented in Table 6. The highest rating overall was found in the use of Zoom as a videoconferencing app (mean 4.14, SD 0.51). The second highest rating overall was found in the use of the avatar in a setting similar to the virtual world (mean 3.83, SD 0.61). The lowest rating overall was found in the use of a phone call as the medium for the alcohol SBI encounter (mean 3.41, SD 0.75).

**Table 6:** Mean scores of Telecom Simulation User Evaluation Survey (N=482).

Telecom Simulation User Evaluation Survey items	Teleconference (Zoom; n=450), mean (SD)	Avatar/virtual world encounter (n=18), mean (SD)	Phone encounter (n=14), mean (SD)
This training mode provided a realistic provider–patient interaction	4.26 (0.70)	3.78 (1.11)	3.21 (1.18)
Experiencing the standardized patient’s voice and facial expressions was important in this interaction	4.46 (0.67)	3.50 (1.15)	3.43 (1.01)
It was just as easy to talk with the patient about substance use in this interactive environment as it would be in real-world training	3.77 (0.93)	3.67 (1.08)	3.00 (1.10)
This mode of interacting was distracting from the content of the conversation	3.79 (0.98)	3.72 (1.17)	3.64 (0.92)
I noticed a delay in response time while using this method of communicating	4.14 (1.06)	3.72 (1.07)	4.00 (0.87)
The standardized patient was skillful and natural in the patient role	4.55 (0.69)	4.11 (1.02)	4.07 (1.14)
Feedback from the standardized patient was informative and useful to me	4.55 (0.63)	4.50 (0.51)	3.86 (1.16)
I prefer this method training to real-life role plays or simulations	3.14 (1.05)	2.83 (1.29)	3.00 (1.03)
Getting set up and started with technology for this simulated SBI^a^ session was easy enough	4.07 (0.91)	3.6 (1.03)	2.36 (1.39)
This mode of experiential training is an expedient method for learning how to conduct a good intervention	4.17 (0.73)	3.78 (0.64)	3.14 (1.29)
I plan to utilize what I have learned from this training in my clinical practice	4.52 (0.60)	4.56 (0.51)	3.93 (0.99)
Overall, the experiential training met or exceeded my expectations	4.30 (0.74)	4.22 (0.87)	3.36 (1.15)
Satisfaction score in the aggregate	4.14 (0.51)	3.83 (0.61)	3.41 (0.75)

^a^SBI: screening and brief intervention.

### Training Satisfaction Survey

Finally, the Training Satisfaction Survey, presented in Table 7, was completed by the trainee immediately following the training (baseline) and 30 days later, at the one-month follow-up time point, resulting in essentially no change over time. The baseline mean score was 4.24 (SD 0.73), and it was unchanged after 30 days, with a mean score of 4.24 (SD 0.73).

In the examination for demographic differences in the pre/posttest mean score analysis (results not shown), we did find significant differences by gender and year in school.

In addition, owing to the significant outcomes across all schools in the pre/post mean scores, we conducted a sensitivity analysis to determine if differences in each school implementation (Table 1) had influenced school performance and if there was any statistical differences between their training outcomes. Although we did show that at the pretest assessment there was a statistical difference between SSC-Knoxville, SLU, and MSU, at the posttest assessment, those differences were resolved. Therefore, the mixed-models factorial ANOVA stratified by institution revealed no statistical difference between the 5 school implementations (data not shown).

**Table 7:** Training Satisfaction Survey baseline and 1-month follow-up scores (n=353).

Training Satisfaction Survey questions	Baseline, mean (SD)	1-month follow-up, mean (SD)
How satisfied are you with the overall quality of this training?	4.26 (0.78)	4.25 (0.77)
How satisfied are you with the quality of the instruction?	4.21 (0.80)	4.22 (0.79)
How satisfied are you with the quality of the training materials?	4.23 (0.78)	4.26 (0.75)
Overall, how satisfied are you with your training experience?	4.28 (0.77)	4.25 (0.81)
Satisfaction aggregate scores	4.24 (0.73)	4.24 (0.73)

## Discussion

### Principal Findings

The development of a cohesive and inclusive Web-based training educational model for health care students is complex. We found that it involves a continuous process that requires detailed feedback mechanisms and flexibility to match emerging needs. The effectiveness of utilizing alcohol SBI in routine clinical practice is not new; however, implementing techniques to assure routine use in practice remains a challenge. In our analysis across these 5 programs, the best training sequence involved a face-to-face presentation at the participant schools, introducing the topic of alcohol SBI and importance of screening for alcohol use; providing direction on how to navigate the course website with details about pre- and postcourse expectations, followed by participation in the alcohol SBI training course; and finally, inclusion of a live session with a standardized patient via videoconferencing for a practice alcohol SBI encounter. However, although this appeared to be the best implementation method based upon satisfaction feedback, in the sensitivity analysis conducted among the 5 programs, we found no significant differences between implementation method and effectiveness of the outcomes. This would suggest that differences in program implementation did not affect the impact of the alcohol SBI Web-based training module.

In the analysis of our results compared with the population of trainees, we did find significant differences by gender and year in school. It appears that women trainees increased their knowledge and skills more, compared with men. Similarly, the students who were in their second year of the program (the clinical year) had higher mean scores at the posttest assessment compared with those in their first year of the program. The gender difference could be because of the greater number of female physician assistant students (70.7%, 341/482), compared with male physician assistant students (29.3%, 141/482) across all participant schools. The fact that students in their second year had higher mean scores would suggest that more experience with patients yields greater knowledge and makes one more comfortable in knowing how to talk with patients.

Finally, it is noted that the student/trainee mean score for the statement regarding the NIAAA low-risk drinking guidelines was low in comparison with other elements in the PRS-provider assessment. The training module instructs the learner about several different types of alcohol screening instruments, one of which is the NIAAA low-risk drinking guidelines. However, the alcohol use guide that the student trainees were instructed to use for the simulated live encounter with the standardized patient used the AUDIT-C (alcohol use disorder identification test consumption) for the instruction on what were low- versus high-risk drinking levels. Although the low-risk drinking guidelines used in the AUDIT were the same as the NIAAA guidelines, the student trainees would most likely not have remembered this in responding this statement on the PRS-provider. This outcome would suggest that this particular question would need to be modified for any future assessments.

In this study, we believed that the development of a Web-based course dedicated to teaching health care practitioners how to hold a conversation about alcohol with a patient/client needed to be engaging and easy to use. We were pleased with the significant difference in change over time for trainee knowledge and skills relevant to conducting an alcohol SBI encounter. The 3 statements that did not achieve significance were, in fact, all pertaining to trainee attitude, which suggests that for this population of physician assistant students, attitude about the importance of discussing alcohol use and screening was already at a high level.

### Simulation and Standardized Patient Encounters

Encountering challenges with deploying and utilizing standardized patient training methods on the Web are not new [11,19]. However, with the advancement of technology-based approaches to address education needs of health care professionals, it is almost an essential tool to meet these needs. In this study, we found that virtual standardized patient encounters did not work well, overall. This finding contends with the larger literature base [10,16]. In theory, moving face-to-face encounters to a virtual environment should be more convenient to use because it minimizes barriers such as cost, access, security, scalability, and flexibility; however, we find that there are several obstacles with transitioning from theory to practice. Research suggests that some of the most pertinent factors to consider when designing/implementing a standardized patient–centered curriculum are location, availability, and cost [20]. Although these factors were accounted for in this study, challenges persisted around (1) standardized patient knowledge of the content matter, (2) standardized patient and student utilization of technology, and (3) coordination of standardized patient encounters. Anecdotal feedback from student participants suggested that the mechanism of the virtual environment was successful. However, navigating through scheduling standardized patient encounters, training, and educating both standardized patients and simulation center instructors was challenging.

The coordination of operating an independent website and employing an established simulation center presented several practical challenges that were difficult to overcome. First, connecting the 2 LMS platforms proved to be problematic and was ultimately abandoned in favor of incorporating all scheduling and video conference aspects into Catalyst Learning Center [18]. Second, scheduling live encounters that worked for the study team, simulation center, and student cohort was complicated, and the arrangement required substantial manual organization. In addition, the challenge to adequately and consistently train a variable pool of standardized patients in a complex behavior change approach was demanding.

The study team provided dedicated instruction to the standardized patients; however, the standardized patients were not alcohol SBI specialists, and the students reported that the standardized patient feedback was often inconsistent with the training content. Even after several modifications based on feedback from the students, study team, and simulation center staff, the standardized patient live-encounter process was challenging to arrange, difficult to manage, and required unsustainable effort to control for human error at various stages. Students generally had positive responses to the live standardized patient encounter and reported that they it beneficial for the training. There is room for improvement in delivering behavior change instruction to standardized patients, and integrating alternate technology will improve the process of accessing standardized patients remotely.

### Program Management Challenges

Coordination among the website developers, simulation center, and research teams required considerable oversight.

#### Individual Schools

Three schools were subcontractors of the grant and were committed from the onset to participation and implementation of the program. Some of the challenges we encountered included getting administrator buy-in, administrator attitudes, and their perception of the importance of the study. In one school, added after the grant commenced, was a new program with only its first cohort of physician assistant students, and creating a structured curriculum for the students and trying to initiate a new training curriculum at the same time was met with resistance. The presence of advocates and/or program champions who could speak about the importance of an alcohol SBI training/curriculum helped facilitate the navigation of the administrative maze. Along the lines of having program advocates within the administration, the literature supports the inclusion of independent faculty development resources to better align desired health care professionals’ training outcomes with training resources [21].

#### Technical Teams

As this was a pilot in which changes were made incrementally to incorporate end-user feedback, the website required constant maintenance and updating to accommodate trainee and researcher expectations. Unanticipated needs and issues resulted in the need for sophisticated updates to the site that were both costly and time intensive for the site developers. As with any Web-based application, Catalyst Learning Center [18] was exposed to security risks. Despite several levels of encryption, security vulnerabilities enabled suspicious activity from outside entities and made it difficult for the users to access the site at times. It is important to note that this is a risk, and there is an increased need to protect Web-based programs of this nature.

#### Learning Management System Communication

Significant challenges arose in communication between the university’s simulation center and the Catalyst website [18]. In the process of addressing *ease of using the site*, we wanted to have the Catalyst site include the ability to schedule the simulation center live standardized patient encounters, where the individual student would view the available standardized patient encounter timeslots and match a standardized patient encounter to when the student had availability. This proved to be beyond the current capacity of both the simulation center and the Catalyst website [18], thus all scheduling for the live standardized patient encounters was done manually.

### Limitations

As this was a pilot program dedicated to the design, development, implementation, and evaluation of a Web-based alcohol SBI training module for physician assistants within an academic program, our primary limitation was the lack of control over how the final training module was implemented within each academic setting. As noted in Table 1, each University or College program implemented the training module in a unique manner, one that was well-suited for their specific academic program. Although this is what we wanted to take place in terms of long-term sustainability of the Web-based training module, this made it challenging for comparability across academic setting. Thus, our results are presented in the aggregate across all academic programs as opposed to a comparison between academic programs.

A second limitation is the ability to generalize our results to other physician assistant programs in the country, again because of unique implementation of the training within each of our participant settings, either University or College. However, although this is seen as a limitation, the sensitivity analysis would suggest that the alcohol SBI training can be implemented in a variety of different physician assistant courses and settings and can be successful in each.

We had some loss of survey response at the 30-day satisfaction survey follow-up period because of an attrition rate of 26.9% (130/482). This again was because of the lack of control over how each faculty member encouraged students to complete the full set of evaluation surveys, although we sent out multiple emails to the students directing them to the Catalyst Learning Center [18] to complete the follow-up satisfaction survey. However, we still retained a 73% completion rate for the satisfaction survey with an overall satisfaction mean score of 4.23, indicating a general satisfaction with the training they received. We conducted an analysis to determine potential differences between those who completed the training and those who did not and found no significant difference in terms of the effectiveness of the outcomes.

### Conclusions

The benefits of employing technology-enhanced learning techniques in health professional training has become widely acknowledged. Utilizing these training methods are not without challenges. We find that employing a combined didactic alcohol SBI training model with virtual standardized patient encounters presented unique challenges in the implementation phase. However, such an approach on the Web is relatively innovative and beneficial for student learning. As placing the training on the Web is a relatively new venture, especially the virtual standardized patient, future studies may explore whether a condensed alcohol SBI training on the Web is beneficial and what, if any, content needs to be expanded, highlighted, or completed in person. Furthermore, we note that as more researchers explore creative ways to educate health care professionals about alcohol SBI techniques, our study provides some insight on how to implement technology-based studies and what pitfalls to avoid.

## Abbreviations

ANOVA: analysis of variance
AUDIT: alcohol use disorders identification test
e-learning: electronic learning
LMS: learning management system
MSU: Missouri State University
NIAAA: National Institute on Alcohol Abuse and Alcoholism
PCP: primary care provider
PRS: Proficiency Rating Scale
SAMHSA: Substance Abuse and Mental Health Services Administration
SBI: screening and brief intervention
SBIRT: screening, brief intervention, and referral to treatment
SC-Columbia: Stephens College, Columbia, Missouri
SC-Knoxville: South College, Knoxville, Tennessee
SLU: Saint Louis University
UMKC: University of Missouri–Kansas City

## References

[1] McKnight-Eily LR, Okoro CA, Mejia R, Denny CH, Higgins-Biddle J, Hungerford D, Kanny D, Sniezek JE (2017). Screening for excessive alcohol use and brief counseling of adults - 17 states and the district of Columbia, 2014. MMWR Morb Mortal Wkly Rep.

[2] Agerwala SM, McCance-Katz EF (2012). Integrating screening, brief intervention, and referral to treatment (SBIRT) into clinical practice settings: a brief review. J Psychoactive Drugs.

[3] Anderson BL, Dang EP, Floyd RL, Sokol R, Mahoney J, Schulkin J (2010). Knowledge, opinions, and practice patterns of obstetrician-gynecologists regarding their patients' use of alcohol. J Addict Med.

[4] Gahagan S, Sharpe TT, Brimacombe M, Fry-Johnson Y, Levine R, Mengel M, O'Connor M, Paley B, Adubato S, Brenneman G (2006). Pediatricians' knowledge, training, and experience in the care of children with fetal alcohol syndrome. Pediatrics.

[5] (2011). SAMHSA-HRSA Center for Integrated Health Solutions.

[6] Babor TF, del Boca F, Bray JW (2017). Screening, brief intervention and referral to treatment: implications of SAMHSA's SBIRT initiative for substance abuse policy and practice. Addiction.

[7] Stoner SA, Mikko AT, Carpenter KM (2014). Web-based training for primary care providers on screening, brief intervention, and referral to treatment (SBIRT) for alcohol, tobacco, and other drugs. J Subst Abuse Treat.

[8] Satre DD, McCance-Katz EF, Moreno-John G, Julian KA, O'Sullivan PS, Satterfield JM (2012). Using needs assessment to develop curricula for screening, brief intervention, and referral to treatment (SBIRT) in academic and community health settings. Subst Abus.

[9] Bernstein E, Bernstein J, Feldman J, Fernandez W, Hagan M, Mitchell P, Safi C, Woolard R, Mello M, Baird J, Lee C, Bazargan-Hejazi S, Broderick K, Laperrier KA, Kellermann A, Wald MM, Taylor RE, Walton K, Grant-Ervin M, Rollinson D, Edwards D, Chan T, Davis D, Marshall JB, Aseltine R, James A, Schilling E, Abu-Hasaballah K, Baumann BM, Boudreaux ED, Maio RF, Cunningham RM, Murrell T, Doezema D, Anglin D, Eliassen A, Martin M, Pines J, Buchanan L, Turner J, D'Onofrio G, Degutis LC, Owens P (2007). An evidence based alcohol screening, brief intervention and referral to treatment (SBIRT) curriculum for emergency department (ED) providers improves skills and utilization. Subst Abus.

[10] Satterfield JM, O'Sullivan P, Satre DD, Tsoh JY, Batki SL, Julian K, McCance-Katz EF, Wamsley M (2012). Using standardized patients to evaluate screening, brief intervention, and referral to treatment (SBIRT) knowledge and skill acquisition for internal medicine residents. Subst Abus.

[11] Lempicki KA, Holland CS (2018). Web-based versus face-to-face interprofessional team encounters with standardized patients. Curr Pharm Teach Learn.

[12] Cook DA, Levinson AJ, Garside S, Dupras DM, Erwin PJ, Montori VM (2010). Instructional design variations in internet-based learning for health professions education: a systematic review and meta-analysis. Acad Med.

[13] Glass JE, Hamilton AM, Powell BJ, Perron BE, Brown RT, Ilgen MA (2015). Specialty substance use disorder services following brief alcohol intervention: a meta-analysis of randomized controlled trials. Addiction.

[14] Fleming M, Olsen D, Stathes H, Boteler L, Grossberg P, Pfeifer J, Schiro S, Banning J, Skochelak S (2009). Virtual reality skills training for health care professionals in alcohol screening and brief intervention. J Am Board Fam Med.

[15] Belfiore MN, Blinka MD, BrintzenhofeSzoc K, Shields J (2018). Screening, brief intervention, and referral to treatment (SBIRT) curriculum integration and sustainability: social work and nursing faculty perspectives. Subst Abus.

[16] Koetting C, Freed P (2017). Educating undergraduate psychiatric mental health nursing students in screening, brief intervention, referral to treatment (SBIRT) using an online, interactive simulation. Arch Psychiatr Nurs.

[17] Langen WH, Hanson D, Fien R, Parkhurst D (2011). The evaluation of physician assistant students' history-taking abilities using actors as standardized patients. J Physician Assist Educ.

[18] (2019). Catalyst Learning Center - Health and Care eLearning.

[19] Zary N, Johnson G, Boberg J, Fors UG (2006). Development, implementation and pilot evaluation of a web-based virtual patient case simulation environment--web-SP. BMC Med Educ.

[20] Alfes CM (2013). Nursing alumni as standardized patients: an untapped resource. Clin Simul Nurs.

[21] West C, Graham L, Palmer RT, Miller MF, Thayer EK, Stuber ML, Awdishu L, Umoren RA, Wamsley MA, Nelson EA, Joo PA, Tysinger JW, George P, Carney PA (2016). Implementation of interprofessional education (IPE) in 16 US medical schools: common practices, barriers and facilitators. J Interprof Educ Pract.

